# The Heretic King: Possible Diagnoses of Pharaoh Akhenaten

**DOI:** 10.7759/cureus.98730

**Published:** 2025-12-08

**Authors:** Matthew D Turner, Kari Schweiger

**Affiliations:** 1 Emergency Medicine, Penn State Health Milton S. Hershey Medical Center, Hershey, USA

**Keywords:** akhenaten, ancient egypt, androgen insensitivity syndrome, klinefelter, medical history

## Abstract

Akhenaten, the heretic pharaoh of Ancient Egypt, is one of the most infamous figures in ancient history. His unprecedented break with over a thousand years of tradition, along with the unusual impression he left on the archaeological record, has prompted generations of Egyptologists to propose theories explaining the bizarre depiction of the pharaoh’s anatomy. In this paper, we review the various diagnoses that have been suggested to account for Akhenaten’s appearance. We ultimately conclude that his distinctive imagery was almost certainly a deliberate propagandistic choice by the monarchy and did not reflect any underlying medical condition.

## Introduction and background

The man whom future generations would know as Akhenaten was born Prince Amenhotep, the second son of Amenhotep III of the 18th Dynasty of Egypt. After decades of foreign invasion, domestic instability, and turmoil, the 18th Dynasty achieved an unprecedented level of stability and control across Egypt, expanding the kingdom’s reach into an empire that stretched into much of the Near East [1]. As the second son, Prince Amenhotep was never expected to succeed his father on the throne. However, the unexpected death of the heir apparent, Prince Thutmose, suddenly placed the young prince in direct line to the throne just a few years before his own father passed. With the death of Amenhotep III, Amenhotep IV gained the throne [1].

Within a few short years, Amenhotep IV unexpectedly made a break with over a thousand years of Egyptian tradition. The new pharaoh rejected the polytheistic system of worship that had been the backbone of Egyptian life for centuries and embraced a new religion, the cult of the Aten [1]. The Aten was the god of the sun, represented by a sun disc with rays that terminated in human hands [2]. The Aten was soon elevated to the exclusion of all other gods [2]. Formerly powerful cults, such as the cult of Amun, which had such an extensive priesthood and controlled so much land and so many resources that it could be considered a “state within a state” [3], were suddenly stripped of power and their temples closed. Even mentions of other gods in imagery and inscriptions were banned, as the kingdom underwent a dramatic excision of Amun from countless statues and temples [2]. As the years passed, the Aten went from the supreme god to the sole god of Egypt; all other gods were “cleansed” from the official record in a massive campaign of “state-sponsored iconoclasm” [1]. As part of this dramatic upheaval, Amenhotep IV renamed himself Akhenaten, meaning “effective for the Aten” [1].

Aten’s kingdom

Akhenaten’s remaking of Egypt went even further. He began a massive building program across the kingdom, erecting new temples dedicated to the Aten. In the fifth year of his rule, he founded a new capital, Akhet-Aten [2], a domain where only the sun disc ruled [1]. Even the very order of nature transformed under Akhenaten: before, pharaohs had professed their role in upholding *maat*, the ancient Egyptian concept of truth, justice, and order. Under Akhenaten, the pharaoh declared that he did not uphold *maat*; he lived on *maat*, effectively becoming a god himself [1]. Akhenaten was not just the sole interpreter of the Aten’s will; the Aten was his father and co-regent over Egypt [1].

As part of this, the royal family became the center of a cult that Egypt had never seen, from imagery of Akhenaten with his wife and daughters to massive statues of Akhenaten and Nefertiti that replaced statues of the old gods [1]. This radical break from 17 centuries of Egyptian history was unprecedented and would earn Akhenaten the ire of subsequent generations [1]. His attempt at imposing a “cultural, religious, and artistic revolution” ultimately ended with his death in 1336 BC at the end of a 17-year reign. After his death, his images were systematically destroyed and his name omitted from the official record, as Egypt rejected his revolution and returned to the old ways [4].

## Review

Imagery of Akhenaten

**Figure 1:**
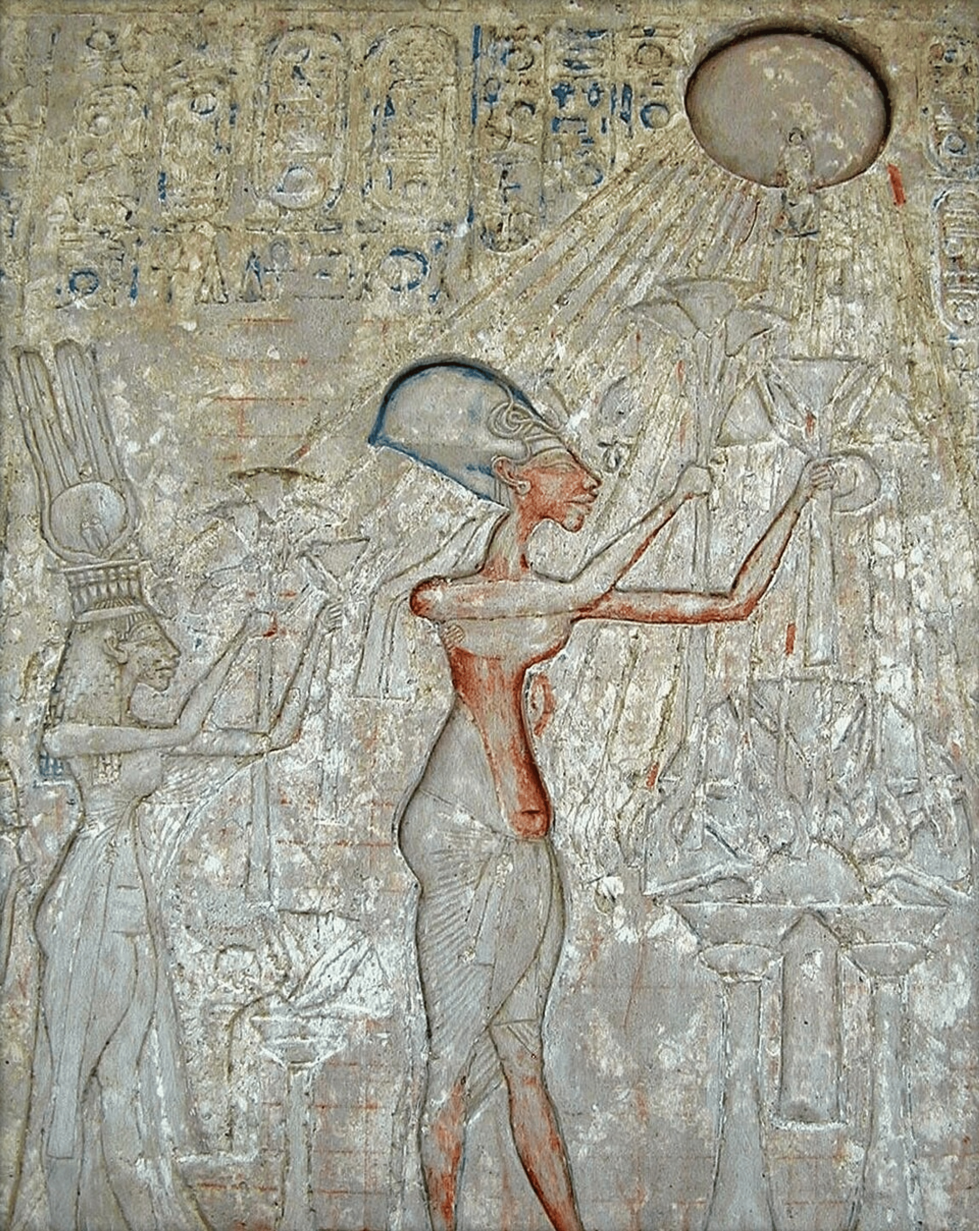
Imagery of Akhenaten praying to the Aten (represented as a sun disc with rays terminating in hands) Note the elongated shape of Akhenaten’s skull, as well as his narrow upper torso, broad hips, gynecomastia, and overall mix of female and male attributes. This work is in the public domain [8].

**Figure 2:**
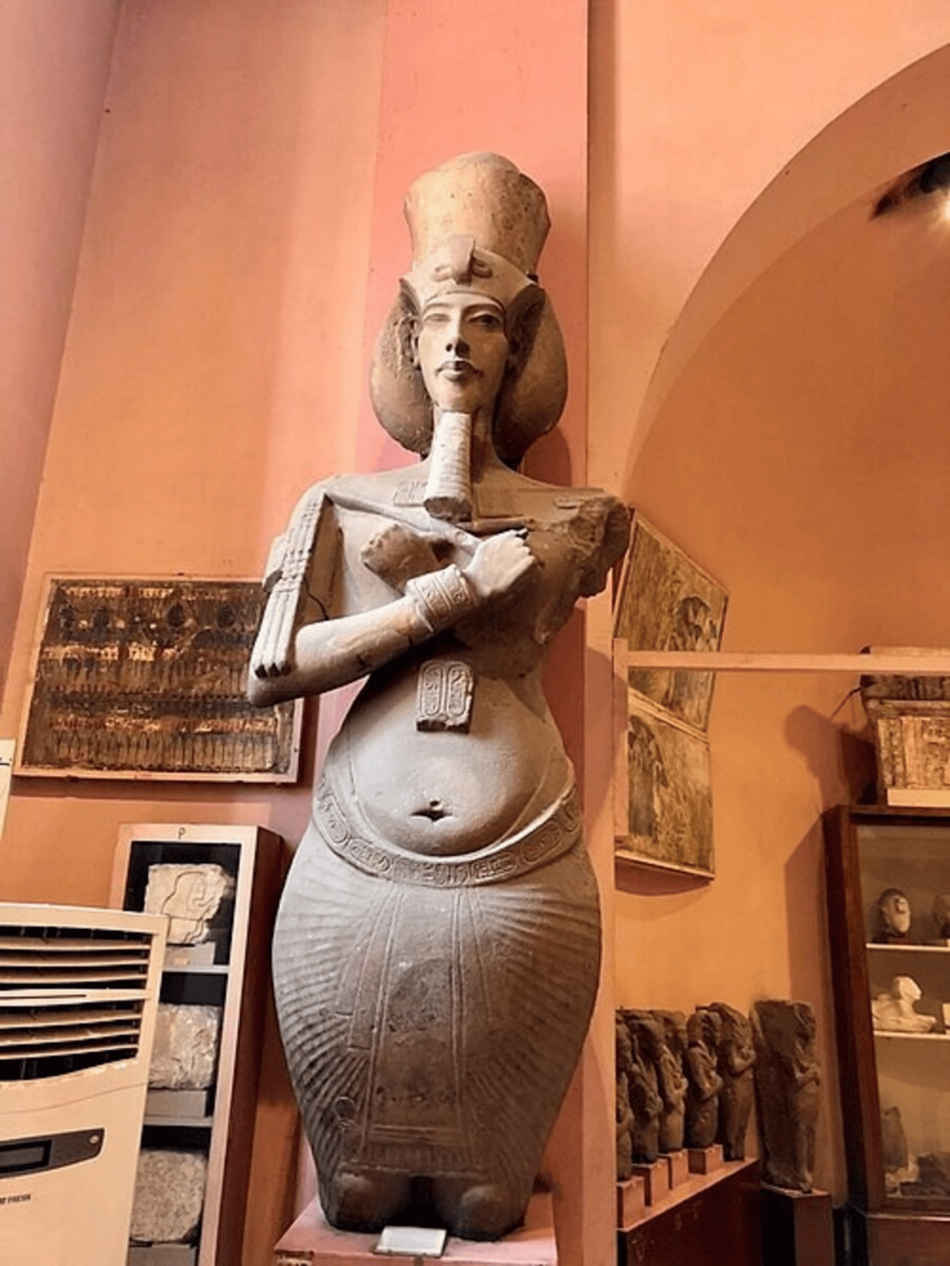
Statue of Akhenaten at the Egyptian Museum in Cairo This image is in the public domain [9].

Despite the attempts of later pharaohs to erase him from the record, Akhenaten’s legacy persists, in large part due to the unusual portraiture that remains of the pharaoh [4]. The imagery is highly unusual and distorted; the pharaoh is depicted with an unnaturally elongated head, a “long, sinewy neck,” and a narrow upper torso, followed by a “distended belly and broad hips; plump legs [that] ended in spindly calves” [1]. Other bizarre features include protruding teeth, large ears, almond-shaped eyes [5], a massive lower jaw [6], and visible gynecomastia [5]. The effect is “floridly androgynous” [5], as though the imagery is meant to combine both masculine and feminine attributes in a way that had never been done before in Egypt [1]. Early Egyptologists were confused by the imagery and were often unsure whether statues of Akhenaten were meant to present a woman or a man [7]. This is demonstrated in Figure 1: a wall mural depicting Akhenaten worshipping the Aten shows a mix of female and male attributes that no pharaoh had ever been depicted with before (Figure 1, Figure 2). The imagery has been compared to that of a “humanoid praying mantis” [5]. Even the pharaoh’s voice was described as strangely soothing and “mellifluous,” as though it were somehow more effeminate [5].

Over the years, Akhenaten’s portraiture has been the subject of fierce debate: was it simply a symbolic choice to represent the new Egyptian faith, or was it actually indicative of an underlying medical diagnosis, one that may have affected other members of the 18th Dynasty? In this review, we discuss the various medical theories that have been proposed and determine which possibility is the most likely.

Physical evidence

In 1907, under the patronage of the American Theodore Davis, archaeologists discovered a tomb in Egypt’s Valley of the Kings. Designated Tomb No. 55, the tomb had clearly already been broken into and then resealed. Unfortunately, the initial description of Tomb No. 55 is “perfunctory,” and the team that discovered it left a poorly written record; however, it is evident that they uncovered a “richly gilded” wooden coffin within the small tomb. Part of the coffin lid had come loose over the intervening centuries, revealing a partially decayed mummy inside [7]. All names on the coffin had been cut away, along with the mummy’s golden portrait mask [7]. A nearby shrine depicted Queen Tiye, mother of Akhenaten, and noted that the shrine had been made for her by her son, but all imagery of Akhenaten and mention of his name had been hacked away [7]. The only exception was several golden bands on the mummy that reportedly bore the name “Akhenaten” [10].

Despite early proposals that Tomb No. 55 was intended for Queen Tiye [7], it appears significantly more likely that the mummy in the tomb (better known as KV55) is Akhenaten himself. A 2010 genetic study of King Tutankhamun, one of the most famous pharaohs of the 18th Dynasty, determined that KV55 was most likely his biological father [11]. This corroborated previous research: in the mid-twentieth century, researchers found that KV55 and Tutankhamun had the same blood group, further suggesting a father/son or brother/brother relationship [12]. Furthermore, a 2016 paper determined that KV55 is the direct descendant of Queen Tiye and mummy CG 61074, widely believed to be the remains of Amenhotep III [12]. Ultimately, while some controversy remains, it appears highly likely that KV55 is indeed the remains of Akhenaten [12].

Early theories

Early Egyptologists were stunned by the portrayal of Akhenaten and attempted to explain the pharaoh’s bizarre appearance with a number of theories that have since been discredited. In 1855, Mariette proposed that Akhenaten had been taken prisoner as a young man and castrated, leading to his androgynous appearance later in life. Several decades later, Lefébure proposed that Akhenaten had actually been a woman disguised as a male pharaoh, citing the earlier example of Hatshepsut, a female ruler of Egypt who had often depicted herself in male attire [7]. Both of these theories appear extremely unlikely and can be discounted, as Akhenaten had at least six children with his wife, Queen Nefertiti [1].

Fröhlich syndrome/adiposogenital dystrophy

After examining the remains of a mummy believed to be that of Akhenaten in 1907, the anatomist Elliot Smith proposed that Akhenaten had suffered from Fröhlich syndrome [7], better known as adiposogenital dystrophy today [6]. Based on his determination that Akhenaten’s remains displayed delayed union of the epiphyses, along with evidence of an “overgrowth of the mandible” and a “slight degree of hydrocephalus,” Smith concluded that the pharaoh’s combination of bony and craniofacial abnormalities could only be explained by an endocrinopathy [7].

Adiposogenital dystrophy is a rare disorder of the endocrine system that results in early-onset obesity in childhood, with significant accumulation of excess adipose tissue within the trunk or face. Patients may experience delayed or incomplete sexual maturation, including delayed puberty and hypogonadism, leading to infertility in both males and females. Cognitive impairment, mood swings, and behavioral issues are also common [13].

While adiposogenital dystrophy may explain Akhenaten’s unusually wide hips and thighs, the pharaoh reportedly fathered at least six children [1], indicating that he had none of the fertility issues expected with this syndrome [5]. It should also be noted that Smith’s 1907 autopsy was affected by several methodological issues: an earlier examination of the mummy had incorrectly determined that it was female, and several of Smith’s close acquaintances had become involved in the increasingly heated debate over the mummy’s identity, leading him to write the report with an uncharacteristic focus on nontechnical language and an obvious desire not to anger either side [7].

Androgen insensitivity syndrome

Androgen insensitivity syndrome has also been proposed as a reason for Akhenaten’s peculiar imagery [5]. This hormonal disorder, most well known for presenting as a female phenotype in patients with XY chromosomes, is most commonly identified in female adolescents presenting with amenorrhea [14]. It is highly unlikely that Akhenaten had the complete form of this endocrinopathy, as imagery from early in his reign depicts him unambiguously as an adult male [5].

A less severe form of this endocrinopathy, known as partial androgen insensitivity syndrome, appears more likely. Patients with this syndrome may experience micropenis, hypospadias, and a feminized appearance in males [14]. However, even milder presentations of this syndrome typically result in infertility [14]; as noted above, Akhenaten showed no evidence of reduced fertility throughout his reign [5].

Klinefelter syndrome

It has been observed by several commentators that the pharaohs of the latter half of the 18th Dynasty, specifically Amenhotep III, Akhenaten, the little-known Smenkhkare, and Tutankhamun, were often depicted with gynecomastia [15], suggesting an underlying familial pathology [11]. While the remains of KV55 and Tutankhamun display no evidence of gynecomastia, KV55 is in poor condition, and Tutankhamun’s mummy lacks an anterior chest wall [11]. This raises the possibility of Klinefelter syndrome.

In his book on ancient Egyptian medicine, Nunn proposes that Akhenaten may have suffered from Klinefelter syndrome [6]. To support this, he highlights the pharaoh’s imagery, which depicts underdeveloped muscularity, particularly in the arms and lower legs, mild obesity, and long lower extremities [6]. In addition, up to 70% of males with Klinefelter syndrome present with gynecomastia [16], a condition that Akhenaten was often depicted with [15]. However, as with the other systemic syndromes noted above, Nunn acknowledges that Akhenaten does not demonstrate any of the reduced fertility that would be expected with Klinefelter syndrome [6].

Aromatase excess syndrome

The persistent gynecomastia depicted in artwork from the 18th Dynasty, while it may be due to a simple form of familial gynecomastia, may also reflect an underlying pathology. By some estimates, at least four generations, from Thutmose I (Akhenaten’s grandfather) to Amenhotep III (Akhenaten’s father), Akhenaten, and then his son Tutankhamun, were depicted with gynecomastia. In 2009, Braverman proposed that the 18th Dynasty may have had aromatase excess syndrome [5].

Aromatase excess syndrome results in estrogen excess [17], notably causing gynecomastia and a eunuchoid habitus in males. Interestingly, Braverman notes that this may explain the effeminate and “mellifluous” voice that Akhenaten was reported to have had [5].

This theory is further supported by depictions of Akhenaten’s family. Affected females may display precocious puberty and macromastia. In one famous image, Akhenaten is shown with his daughter, Princess Meketaten, who, despite being only five to seven years old, is depicted with unusually enlarged breasts and prominent hips. Other images of the pharaoh’s daughters, despite their young ages, are similarly portrayed [5].

While aromatase excess syndrome produces gynecomastia in males, it does not result in significant hypogonadism [17], which may explain Akhenaten’s apparent fertility [5]. Although Akhenaten’s mummified remains are in poor condition, his son, Tutankhamun, was noted to have normal genitals, with no evidence of hypogonadism [11].

However, it should be noted that there are no documented cases of adult-onset gynecomastia in aromatase excess syndrome; it consistently develops between seven and 14 years of age [17]. While the historical record is silent on the exact age at which Akhenaten came to the throne, imagery from the early years of his reign depicts him as an adult male, with no evidence of gynecomastia or the other unusual attributes seen in his later portraiture [5]. Aromatase excess syndrome was only recently identified, and there are still only a handful of cases described in the literature [17]. While it is possible that Akhenaten experienced an unusually late presentation of this disorder, the current body of evidence for aromatase excess syndrome does not support this explanation.

Marfan syndrome

As noted earlier, Elliot Smith’s 1907 autopsy of KV55 was plagued by several issues. Smith initially claimed that the skull showed evidence of hydrocephalus, with a strangely enlarged and flattened appearance [7]. Because this could indicate dolichocephaly, Marfan syndrome has been proposed as a possible explanation for the pharaoh’s unique presentation [11].

Marfan syndrome is an autosomal dominant condition that primarily affects fibrillin-1, a major component of the extracellular matrix and an important element of the body’s connective tissues. Patients with Marfan syndrome most often present with aortic root aneurysms, ocular abnormalities, an elevated risk of aortic dissection, and overgrowth of the long bones of the skeleton [18]. Dolichocephaly is also a common feature [11].

**Figure 3:**
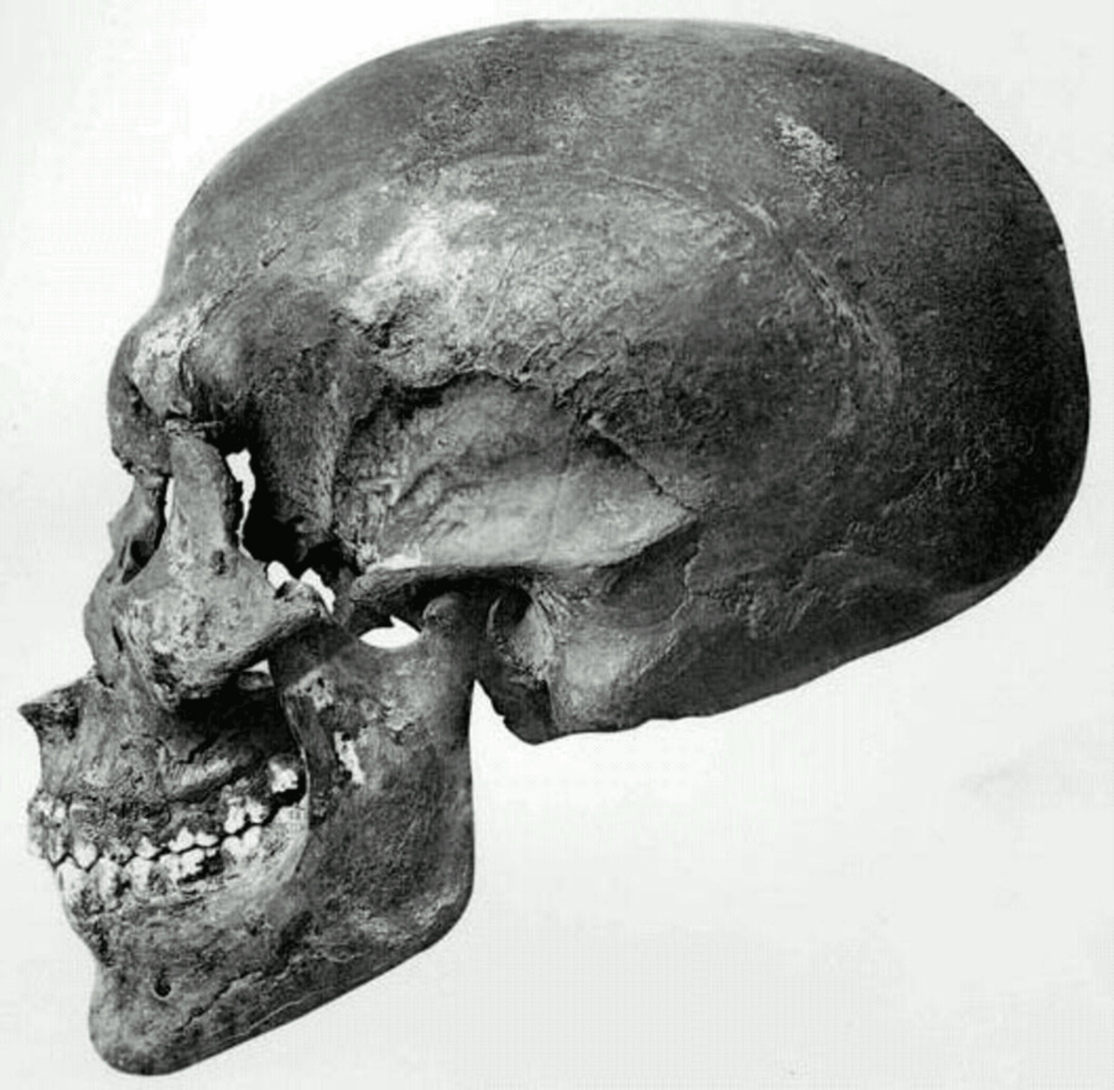
Skull of KV55 This image is in the public domain [10].

However, following the initial 1907 autopsy, later researchers found that Akhenaten does not demonstrate an increased endocranial volume or evidence of dolichocephaly. A 1966 study found that the KV55 mummy had a normal endocranial volume (Figure 3). While slightly brachycephalic, it still fell within the normal range for contemporary adults [19]. Akhenaten’s cephalic index of 81.0, with no evidence of premature closure of the skull sutures, indicates that a diagnosis of Marfan syndrome is extremely unlikely. Akhenaten’s son, Tutankhamun, had a similar cephalic index of 83.9, further supporting this finding [11].

Antley-Bixler Syndrome

A 2009 study noted that Akhenaten’s imagery, with a prominent chin, long neck, and elongated occiput, was consistent with dolichocephaly. These features were also depicted in images of his wife, Nefertiti, as well as in images of his daughters, suggesting a possible craniosynostosis syndrome [5]. The researchers proposed Antley-Bixler syndrome, a rare genetic disorder that causes abnormal formation of the cranial bones [5]. One variant of this syndrome can also impair steroidogenesis, resulting in endocrine abnormalities that may explain the gynecomastia and feminization seen in Akhenaten’s imagery [5].

Notably, this paper states that Akhenaten’s mummy has never been located [5]. While some controversy still exists, the evidence strongly supports that the KV55 mummy is Akhenaten [11]. Additionally, both KV55 and Tutankhamun show no evidence of premature closure of the skull sutures or of any craniosynostosis that would indicate Antley-Bixler syndrome or any other underlying systemic pathology [11].

Propaganda

Ultimately, the most likely conclusion is that the imagery of Akhenaten was a deliberate and powerful use of propaganda. Akhenaten likely combined masculine and feminine features in depictions of himself to convey that, like the Aten, he embodied both male and female attributes, the sole creator of the universe [1]. The uncanny imagery may also have served to terrify and intimidate [1].

Nestor L’Hôte, a prominent Egyptologist of the early 19th century, emphasized the striking impact of Akhenaten’s unique imagery, even thousands of years after his death. Many Egyptologists have since suggested that this effect was intentional. By breaking away from centuries of tradition and adopting “attention-grabbing new imagery,” the pharaoh’s iconography was designed to shock and overwhelm viewers, asserting his authority [20]. In a largely illiterate society [1], the symbology of the king’s imagery was not only literal but also a sophisticated means of communicating complex propaganda [20]. As Wilkinson notes, the overall effect of Akhenaten’s distorted appearance, “especially when multiplied over and over again at a colossal scale in the harsh, raking light of the open court, was both frightening and surreal” [1]. Fear of the pharaoh’s power was particularly important; one surviving letter from a vassal reads, “I am the dirt beneath the sandals of the king, my lord. My lord is the sun who comes forth over all lands day by day…” [1].

More broadly, Egyptian portraiture aimed to convey ideals or classify subjects, rather than provide strictly realistic representations [20]. The ancient Egyptians were masters of propaganda, skilled at recording history as “what they wished it to be” [1]. Pharaohs, in particular, were depicted in highly symbolic and idealized ways. Royal imagery was intended as “an ideal substitute of the King’s essence, a symbol of kingship rather than the portrait of a man” [20].

Although depictions of medical conditions, such as dwarfism, male gynecomastia, clubfoot, and hernias, appear in ancient Egyptian art [6], they are largely confined to medical contexts and portray commoners. It is highly unlikely that a ruling pharaoh would publicly display such weaknesses. Even Tutankhamun, known for being depicted with a cane [21], seems to have had this imagery only in his tomb. Canes were also symbols of nobility [21], so it is uncertain whether this reflected a medical condition. Even if the cane was related to avascular necrosis, as proposed by Hawass in 2010 [11], it was confined to private tomb imagery and not presented publicly.

Akhenaten was not the first pharaoh to use unconventional imagery to convey a message. Senusret III of the 12th Dynasty, known for his powerful centralized rule, depicted himself with macrotia in statues placed throughout his kingdom. By showing enlarged ears, he symbolically warned his subjects that he was all-hearing and all-knowing [1]. These depictions were not realistic but symbolic, emphasizing the king’s strength and influence while warning against rebellion [1]. Similarly, Hatshepsut, a female pharaoh, was often depicted as male to strengthen her claim to the throne, blending masculine and feminine traits much like Akhenaten later did [1].

Even the gods were not exempt from symbolic depictions with both masculine and feminine characteristics. As Codaccioni notes in a 2013 review, several male deities were shown with enlarged breasts, symbolizing fertility linked to the flooding of the Nile [22].

Furthermore, imagery of Akhenaten from the early years of his reign, when he still ruled as Amenhotep IV, follows traditional Egyptian style, depicting him with a “relatively normal face and physique.” It was only after he embraced the cult of the Aten that his imagery changed [5], further supporting the conclusion that these depictions were propagandistic rather than reflective of a medical condition.

This interpretation is corroborated by physical evidence. The initial 1907 autopsy of KV55 was rushed and flawed [7]; a 1966 examination found the remains to fall within the typical range for “bodily physique and proportions” [19]. More recent studies have likewise found no evidence of gross syndromes or severe abnormalities in the pharaoh’s remains that would indicate a systemic medical condition [11].

## Conclusions

The striking imagery associated with Pharaoh Akhenaten has puzzled Egyptologists for decades. Although the depictions have prompted numerous medical hypotheses over the years, the available physical evidence strongly suggests that this imagery was intentionally designed as a form of religious and political propaganda.
